# The timing of complementary feeding in preterm infants and the effect on overweight: study protocol for a systematic review

**DOI:** 10.1186/s13643-016-0324-3

**Published:** 2016-09-02

**Authors:** Karin M. Vissers, Edith J. M. Feskens, Johannes B. van Goudoever, Arieke J. Janse

**Affiliations:** 1 Department of Pediatrics, Hospital Gelderse Vallei, Ede, The Netherlands; 2 Division of Human Nutrition, Agrotechnology and Food Sciences Group, Wageningen University, Wageningen, The Netherlands; 3 Department of Pediatrics, Emma Children’s Hospital, Academic Medical Center and VU University Medical Center Amsterdam, Amsterdam, The Netherlands

**Keywords:** Preterm infants, Complementary feeding, Obesity, Overweight

## Abstract

**Background:**

In term infants, there is evidence that early complementary feeding is a risk factor for childhood obesity. Therefore, timely introduction of complementary feeding during infancy is necessary. The World Health Organization (WHO) and European Society for Paediatric Gastroenterology Hepatology and Nutrition (ESPGHAN) both developed recommendations for the start of complementary feeding for term-born infants. However, these guidelines cannot be directly translated to preterm infants. Recent literature looking at the introduction of complementary feeding in preterm infants gives contrasting information. Given these contrasting reports on the introduction of solid foods in premature born infants, a systematic review is needed. The primary objective of this study is to analyze the effect of the time starting complementary feeding on overweight (including obesity) in preterm infants.

**Methods:**

An electronic systematic literature search with pre-defined terms will be conducted in Cochrane, PubMed, EMBASE, Web of Science, Scopus, and CINAHL. There will be no restriction for time period. Primarily, data from randomized controlled trials (RCTs) will be included in this systematic review. Search terms will include preterm infants, complementary feeding, overweight, and their synonyms. Article selection, including risk of bias assessment, will be performed by three reviewers independently. Body mass index standard deviation score (BMI-SDS or BMI-Z-score) will be used to compare studies. The consistency of results across the studies will influence the decision whether or not to combine results in a meta-analysis. Studies that cannot be included in the meta-analysis will be described in a narrative analysis.

**Discussion:**

This systematic review will give an overview of the existing knowledge on the timing of complementary feeding in preterm infants and the effect on overweight. It will form a basis for future guidelines for complementary feeding for preterm infants.

**Systematic review registration:**

PROSPERO CRD42015014215

**Electronic supplementary material:**

The online version of this article (doi:10.1186/s13643-016-0324-3) contains supplementary material, which is available to authorized users.

## Background

Complementary feeding is defined as the introduction of non-(breast)milk foods or nutritive liquids when milk alone is no longer sufficient to meet all nutritional requirements of infants. In this period, there is a gradual transition to eating family foods [1, 2]. Complementary feeding is associated with major changes in both macronutrient and micronutrient intake. Timely introduction of complementary feeding during infancy is necessary for both nutritional and developmental reasons [2]. In healthy term-born infants living in Europe, the recommendations for the age at which complementary feeding should be introduced are based on considerations on the optimal duration of exclusive breastfeeding. A World Health Organization (WHO)-commissioned systematic review concluded that there were no differences in growth between infants exclusively breast-fed for 3–4 months versus 6 months. Therefore, the WHO recommends mothers worldwide to exclusively breastfeed infants for the child’s first 6 months to achieve optimal growth, development, and health. Thereafter, they should be given nutritious complementary foods and continue breastfeeding up to the age of 2 years or beyond [3–5]. The European Society for Paediatric Gastroenterology Hepatology and Nutrition (ESPGHAN) Committee recommended that the introduction of complementary feeding should not be before 17 weeks but should not be delayed beyond 26 weeks of age, acknowledging exclusive or full breastfeeding until 6 months as a desirable goal [6].

In term infants, early complementary feeding may be a risk factor for childhood obesity. A systematic review and meta-analyses of risk factors for childhood overweight identifiable during infancy by Weng et al. found some evidence supporting the early introduction of solid foods as a risk factor for later overweight [7]. However, a systematic review determining whether the timing of introducing solid foods is associated with obesity in infancy and childhood by Moorcroft et al. did not find a clear association between the timing of introducing solid foods and obesity in infancy and childhood [8]. Furthermore, Pearce et al. showed in a recent systematic review in 2013 about the timing of introduction of complementary feeding in term infants, and the risk of childhood obesity concluded that the timing of complementary foods has no clear association with childhood obesity, although very early introduction of solid foods (≤4 months of age) may result in an increase in childhood BMI [9].

These reviews and recommendations however concern healthy term-born infants, and results cannot be translated to preterm infants. Preterm infants are a heterogeneous population because their gestational age at birth could vary between 23 to 36 weeks. In the Netherlands, the incidence of preterm birth (<37 weeks gestational age) is 7.7 % and very preterm birth (<32 weeks gestational age) 1.3 % [10]. Multiple factors may be associated with obesity in preterm infants, such as the timing of complementary feeding, rapid weight gain, a higher birth weight, a longer gestational age, maternal over-nutrition resulting in a phenotype that is characteristic of metabolic syndrome, and epigenetic factors [11, 12]. Yet, limited evidence is available about the optimal age of solid food introduction in preterm infants and implications for both short- and long-term health and in particular obesity. A study by Casey et al. showed in a longitudinal cohort study that high weight gain in the first year of life is an important predictor of the development of obesity at the age of 8 years in low birth weight preterm infants [13]. A study by Jingxion et al. showed that the introduction of semi-solid foods before the age of 4 months in both term and preterm children resulted in a higher prevalence of overweight at the age of 2 years in comparison to introduction after 4 months of age in children visiting community health centers. In this study, the overall prevalence of overweight was 4.7 % [14].

Observational studies in developed countries have found that solid foods have been introduced to the majority of the preterm infants prior to 4 months of corrected age [15, 16]. The age at which solid foods are introduced is thought to be crucial for infants learning to eat. Infants who lack the opportunity to practice various feeding skills at appropriate ages appear to be at risk of feeding problems later on. A recent review by Palmer et al. outlined the challenges of introducing solid foods to preterm infants and evaluated the benefits and risks. According to this review, starting solid foods in every premature infant should be individualized taking into account infants’ gestational age at birth, early nutrition intake, and current nutritional status and requirements, as well as developmental progress and readiness. The factors that should be taken into account are the risk of developing obesity, increased gut permeability of the preterm infants leading to the risk of allergies, immaturity of the kidney function of the preterm infants, and the increased risk of hospitalization from infections [17]. King has presented guidelines for preterm infants. She suggests that most preterm infants may be ready for solid foods between 5 and 8 months of uncorrected age, provided that the infant is at least 3 months of corrected age (gross motor development should enable safe eating). Moreover, infants who do not start to eat solid foods at an appropriate age can have feeding problems later in life. Hence, according to the review by King, the introduction of solid food assists speech development [17, 18].

Given these contrasting reports on the introduction of solid foods in premature born infants, a systematic review is needed. The primary objective of this study is to analyze the effect of the timing of the start of complementary feeding in preterm infants on overweight.

## Methods

This protocol follows the Preferred Reporting Items for Systematic Reviews and Meta-Analyses Protocol 2015 (PRISMA-P) (see Additional file 1) [19].

### Criteria for considering studies for this review

#### Types of studies

Primarily, data from randomized controlled trials (RCTs) will be included in this systematic review. In addition, cohort studies (both prospective and retrospective, with and without control group) will also be included. Case reports will be excluded. Given the expected clinical and methodological heterogeneity, there will be no minimum length of follow-up.

#### Types of participants

We will include studies examining premature infants, defined as born before 37 weeks of gestational age.

#### Types of interventions

Timing of complementary feeding is a process of introducing any non-breast milk foods or nutritive liquids,when breast or formula milk alone is no longer sufficient to meet all nutritional requirements [1]. Early start of complementary feeding is defined as starting before 15 weeks corrected gestational age. Intermediate start of complementary feeding is defined between 15 to 23 weeks corrected gestational age, and late start of complementary feeding is defined as after 24 weeks corrected gestational age.

#### Primary outcomes

For the primary outcome of this review, we measured height and weight. Studies with self-reported height and weight will not be included. To account for sex- and age-related changes over time, body mass index standard deviation score (BMI-SDS or BMI-Z-score) and percentage overweight will be used to compare studies in the results section of this review. When BMI-SDS or BMI-Z-scores are not reported, these will be calculated or extracted from data given in the report. If calculation is not possible, the authors will be contacted. If BMI-SDS or BMI-Z-scores are not available anyhow, the change in BMI will be used as the second best outcome variable.

### Search methods for identification of studies

#### Electronic searches

An electronic systematic literature search will be conducted in Cochrane, PubMed, EMBASE, Web of Science, Scopus, and CINAHL. The search will be done using pre-defined search terms, including preterm infants, complementary feeding, overweight, and their synonyms as noted in the syntax (see Additional file 2). The search will be limited to articles in English, French, German, Spanish, and Dutch. There will be no restriction for time period. We will exclude animal studies, expert opinions, letters to the editor, protocols, and secondary analysis.

#### Searching other resources

Additionally a related article search will be performed in PubMed and after screening the full-text articles cross-references will be checked for relevant references. Furthermore, ClinicalTrials.gov, The European Union Clinical Trials Register, and the Netherlands Trial Register will be searched.

### Data collection and analysis

#### Selection of studies

After electronic searching, the records will be moved to a database set-up by EndNote software. EndNote will be used to remove all duplicates automatically. Afterwards, duplicates will be removed manually as well. The retrieved studies will be screened independently by three reviewers, namely KV, AJ, and EF. First, the titles of the retrieved studies will be screened and irrelevant studies will be excluded. Subsequently, abstracts of the remaining studies will be screened as well. Articles will be included if it is written in English, French, German, Spanish, or Dutch, if it is a primary study design, and if the study matches both domain (infants born preterm) and determinant (the start of complementary feeding). After screening for title and abstract, full-text papers will be obtained by one member of the review team. Studies will be excluded if no full-text version is available, after having attempted to contact the author. The full-text articles will then be assessed on in- and exclusion criteria by three of the members of the review team. Any discrepancies between the three reviewers in this process will be discussed. If necessary, a fourth person, namely JvG, will be consulted. A flow diagram will be used to summarize the study selection processes.

### Data extraction and management

We will develop a data extraction sheet and pilot test on ten randomly selected studies and refine it accordingly. KV will extract the data from the included studies, and AJ will check the extracted data. Information on the descriptive and quantitative characteristics of each included study will include the following:

1. Characteristics of the study: study design, country, setting, sample size, number of centers, duration of follow-up, source of funding
2. Publication details: year of publication, language, publication status
3. Information of domain and population: preterm infants (definition, age, mean age, in- and exclusion criteria), number of participants, sex, ethnicity
4. Information of determinant: complementary feeding (definition, time of starting complementary feeding (corrected or uncorrected gestational age), type of complementary feeding, duration of complementary feeding), number of intervention/controls
5. Information of outcome: primary and secondary outcomes, definition, measurement methods, time-point(s), summary of the outcome, sample size, missings, estimated effect, subgroup analysis

#### Assessment of risk of bias in included studies

Assessing the risk of bias in the included studies will be performed according to the Cochrane Risk of Bias Tool [20], to assess the risk of bias in randomized controlled trials, and will be supplemented with items extracted from the Newcastle-Ottawa quality assessment tool [21] and from the Agency for Healthcare Research and Quality (AHRQ) publication of Viswanathan et al. [22]. The tool consists of items that cover five domains of bias, namely selection, performance, attrition, detection, and reporting, as well as an “other bias” category to capture other potential threats to validity (see Additional file 3). Selection bias will include information about the sequence generation and allocation concealment. Performance bias will include information about the blinding of the participants and researchers and the detection bias about the blinding of the outcome assessment. The attrition bias will include information about incomplete outcome data and the reporting bias about the selective outcome reporting and the reporting of the prespecified outcomes. The risk of bias for each study will be assessed by three reviewers, namely KV, AJ, and EF. Disagreements will be resolved by consensus or by a fourth team member, namely JvG, if needed. The reviewers will not be blinded with respect to study authors, institution, or journal. The risk of bias will be scored as “low risk of bias,” “unclear risk of bias,” and “high risk of bias,” using the questions of the Cochrane Collaboration’s tool for assessing risk of bias [20]. The likelihood of publication bias will be assessed by using a funnel plot of the mean differences for asymmetry. Since graphical evaluation can be subjective, we will also conduct statistical test using the Egger regression test. The results of the risk of bias assessment will be presented in the final report, and the reasons for exclusion will be explained. 

#### Measures of treatment effect

Our primary outcome could be reported in BMI-SDS or BMI-Z-score. When BMI-SDS or BMI-Z-scores are not reported, these will be calculated or extracted from data given in the report. If calculation is not possible, the authors will be contacted. If BMI-SDS or BMI-Z-scores are not available anyhow, the absolute change in BMI will be used as the second best outcome variable. Furthermore, the time of starting complementary feeding will be compared between the included studies.

#### Dealing with missing data

In case of incomplete date, two attempts will be made to contact the corresponding author by email. If the authors provide no additional information, a decision will be taken by at least two authors on the inclusion of the study in the final analysis. If feasible, we will incorporate loss-to-follow-up data. These results will be stated.

#### Data synthesis

A flow diagram illustrates the literature search and article selection progress. A table will provide an overview of the articles that are included and will summarize their characteristics. The excluded articles will be discussed in the text briefly. If the available data will allow us to do so, we will also aim to give summary measures.

#### Assessment of heterogeneity

By providing a systematic table, by using the Cochrane Risk of Bias Tool, we will detect possible sources of clinical, methodological, or statistical heterogeneity. The *I*^2^ statistic will be used to assess whether the observed variability in study results is greater than expected to occur by chance (statistical heterogeneity). Thresholds for the interpretation of *I*^2^ will be according to the guidance for Cochrane reviews [20]:

- 0–40 %: might not be important
- 30–60 %: may represent moderate heterogeneity*
- 50–90 %: may represent substantial heterogeneity*
- 75–100 %: considerable heterogeneity*

*The importance of the observed value of *I*^2^ will depend on the magnitude and direction of the effects and the strength of evidence for heterogeneity.

The consistency of results across the studies will influence the decision whether or not to combine results in a meta-analysis. Studies that cannot be included in the meta-analysis will be described in a narrative analysis. If the available data will allow us to do so, we will also aim to give summary measures. For dichotomous outcomes, risk ratio, risk difference, or odds ratio with 95 % confidence interval or frequencies with percentage will be used for pooling. For continuous outcomes, means with standard deviation or mean differences and 95 % confidence intervals will be used for pooling. Standardized mean differences will be used for pooling where continuous outcomes are reported using different measurements or scales. If possible, transformation of data to allow analyses with mean differences will be made. Data will be presented in forest plots. R version 2.15.1 with the metafor package will be used for meta-analysis.

#### Subgroup analysis and investigation of heterogeneity

When sufficient data is available, we will perform subgroup analysis for children small for gestational age, defined as a birth weight below the 10th percentile for gestational age, versus children appropriate for gestational age [23]. We also aim to perform subgroup analysis for children born before 30-week gestational age and after 30-week gestational age.

#### Sensitivity analysis

We will conduct sensitivity analyses to establish if the results are influenced by statistical diversities. If severe heterogeneity is present, analyses both with and without outlying trails will be performed. Furthermore, reanalyzing of the data will be performed in case of inconsistencies of the results, large numbers of missing data in studies, or if multiple statistical approaches are applicable.

## Conclusion/discussion

The lack of guidelines and contrasting information in the recent literature for the timing of complementary feeding gives us the need for a systematic review. In our knowledge, this systematic review will be the first to analyze the effect of the time when complementary feeding is started on overweight in preterm infants. By specifying the optimal age of the timing of complementary feeding, this systematic review will form a basis for future guidelines for complementary feeding for preterm infants. Taking into account the implications on obesity, the incidence of overweight in a vulnerable group as preterm infants will be prevented.

## Additional files

Additional file 1: Preferred Reporting Items for Systematic Reviews and Meta-Analyses Protocol 2015 (PRISMA-P). (DOC 83 kb) Additional file 2: Search strategy. Detailed description of search strategy using the following electronic databases: PubMed, Cochrane Library, EMBASE, CINAHL, Web of Science, Scopus, and CINAHL. (DOCX 29 kb) Additional file 3: Risk of Bias Assessment Tool. Risk of Bias Assessment Tool form that will be used to assess the risk of bias in included studies in this systematic review. (XLSX 14 kb)

## Abbreviations

AHRQ: Agency for Healthcare Research and Quality
BMI: Body mass index
BMI-SDS: Body mass index standard deviation score
ESPGHAN: European Society for Paediatric Gastroenterology Hepatology and Nutrition
PRISMA-P: Preferred Reporting Items for Systematic Reviewsand Meta-analyses Protocol
RCT: Randomized controlled trials
WHO: World Health Organization
