# Supplementary material for: The timing of complementary feeding in preterm infants and the effect on overweight: study protocol for a systematic review
Source: Syst Rev. 2016 Sep 2;5(1):149. doi: 10.1186/s13643-016-0324-3 (PMC5010712; doi:10.1186/s13643-016-0324-3)
Supplement: Additional file 1: — Preferred Reporting Items for Systematic Reviews and Meta-Analyses Protocol 2015 (PRISMA-P). (DOC 83 kb) [file 13643_2016_324_MOESM1_ESM.doc]

**PRISMA-P (Preferred Reporting Items for Systematic review and Meta-Analysis Protocols) 2015 checklist: recommended items to address in a systematic review protocol***

| Section and topic | Item No | Checklist item |
| --- | --- | --- |
| ADMINISTRATIVE INFORMATION | | |
| Title: |  |  |
| Identification | 1a | Identify the report as a protocol of a systematic review  **See page 1, line 2** |
| Update | 1b | If the protocol is for an update of a previous systematic review, identify as such  **Not applicable** |
| Registration | 2 | If registered, provide the name of the registry (such as PROSPERO) and registration number  **See page 2, line 44** |
| Authors: |  |  |
| Contact | 3a | Provide name, institutional affiliation, e-mail address of all protocol authors; provide physical mailing address of corresponding author  **See page 1, lines 4-13** |
| Contributions | 3b | Describe contributions of protocol authors and identify the guarantor of the review  **See page 13, lines 276-280 (Authors’ contributions)** |
| Amendments | 4 | If the protocol represents an amendment of a previously completed or published protocol, identify as such and list changes; otherwise, state plan for documenting important protocol amendments  **Not applicable** |
| Support: |  |  |
| Sources | 5a | Indicate sources of financial or other support for the review  **Not applicable** |
| Sponsor | 5b | Provide name for the review funder and/or sponsor  **See page 13, lines 266-267** |
| Role of sponsor or funder | 5c | Describe roles of funder(s), sponsor(s), and/or institution(s), if any, in developing the protocol  **Not applicable** |
| INTRODUCTION | | |
| Rationale | 6 | Describe the rationale for the review in the context of what is already known  **See page 3-5, lines 47-103** |
| Objectives | 7 | Provide an explicit statement of the question(s) the review will address with reference to participants, interventions, comparators, and outcomes (PICO)  **See page 5, lines 104-106** |
| METHODS | | |
| Eligibility criteria | 8 | Specify the study characteristics (such as PICO, study design, setting, time frame) and report characteristics (such as years considered, language, publication status) to be used as criteria for eligibility for the review  **See page 5-6, lines 112-126** |
| Information sources | 9 | Describe all intended information sources (such as electronic databases, contact with study authors, trial registers or other grey literature sources) with planned dates of coverage  **See page 6-7, lines 136-138 and 143-146** |
| Search strategy | 10 | Present draft of search strategy to be used for at least one electronic database, including planned limits, such that it could be repeated  **See page 6-7, lines 138-142** |
| Study records: |  |  |
| Data management | 11a | Describe the mechanism(s) that will be used to manage records and data throughout the review  **See page 7, lines 162-176** |
| Selection process | 11b | State the process that will be used for selecting studies (such as two independent reviewers) through each phase of the review (that is, screening, eligibility and inclusion in meta-analysis)  **See page 7, lines 148-161** |
| Data collection process | 11c | Describe planned method of extracting data from reports (such as piloting forms, done independently, in duplicate), any processes for obtaining and confirming data from investigators  **See page 7-8, lines 148-164** |
| Data items | 12 | List and define all variables for which data will be sought (such as PICO items, funding sources), any pre-planned data assumptions and simplifications  **See additional file 2** |
| Outcomes and prioritization | 13 | List and define all outcomes for which data will be sought, including prioritization of main and additional outcomes, with rationale  **See page 9, lines 197-203** |
| Risk of bias in individual studies | 14 | Describe anticipated methods for assessing risk of bias of individual studies, including whether this will be done at the outcome or study level, or both; state how this information will be used in data synthesis  **See page 9-10, lines 177-196** |
| Data synthesis | 15a | Describe criteria under which study data will be quantitatively synthesised  **See page 9, lines 209-213** |
|  | 15b | If data are appropriate for quantitative synthesis, describe planned summary measures, methods of handling data and methods of combining data from studies, including any planned exploration of consistency (such as I^2^, Kendall’s τ)  **See page 10, lines 214-228** |
|  | 15c | Describe any proposed additional analyses (such as sensitivity or subgroup analyses, meta-regression)  **See page 11, lines 236-245** |
|  | 15d | If quantitative synthesis is not appropriate, describe the type of summary planned  **See page 10-11, lines 228-232** |
| Meta-bias(es) | 16 | Specify any planned assessment of meta-bias(es) (such as publication bias across studies, selective reporting within studies)  **See page 9, lines 192-196** |
| Confidence in cumulative evidence | 17 | Describe how the strength of the body of evidence will be assessed (such as GRADE)  **Not applicable** |

*** It is strongly recommended that this checklist be read in conjunction with the PRISMA-P Explanation and Elaboration (cite when available) for important clarification on the items. Amendments to a review protocol should be tracked and dated. The copyright for PRISMA-P (including checklist) is held by the PRISMA-P Group and is distributed under a Creative Commons Attribution Licence 4.0.**

*From: Shamseer L, Moher D, Clarke M, Ghersi D, Liberati A, Petticrew M, Shekelle P, Stewart L, PRISMA-P Group. Preferred reporting items for systematic review and meta-analysis protocols (PRISMA-P) 2015: elaboration and explanation. BMJ. 2015 Jan 2;349(jan02 1):g7647.*
