# Supplementary material for: The timing of complementary feeding in preterm infants and the effect on overweight: study protocol for a systematic review
Source: Syst Rev. 2016 Sep 2;5(1):149. doi: 10.1186/s13643-016-0324-3 (PMC5010712; doi:10.1186/s13643-016-0324-3)
Supplement: Additional file 2: — Search strategy. Detailed description of search strategy using the following electronic databases: PubMed, Cochrane Library, EMBASE, CINAHL, Web of Science, Scopus, and CINAHL. (DOCX 29 kb) [file 13643_2016_324_MOESM2_ESM.docx]

# Search Strategy

PUBMED

| **Search** | **Terms** |
| --- | --- |
| #1 | Search (((((((Preterm[Title/Abstract]) OR Prematurely[Title/Abstract]) OR Premature[Title/Abstract]) OR Prematurity[Title/Abstract]) OR birth, premature[MeSH Terms]) OR infant, premature[MeSH Terms]) OR infant, premature, diseases[MeSH Terms]) OR Infant, Extremely Premature[MeSH Terms] |
| #2 | Search (((((((((((((((((((((((((((((((Baby[Title/Abstract]) OR Babies[Title/Abstract]) OR Neonate[Title/Abstract]) OR Neonates[Title/Abstract]) OR Infant[Title/Abstract]) OR Infants[Title/Abstract]) OR Infancy[Title/Abstract]) OR Child[Title/Abstract]) OR Children[Title/Abstract]) OR Childhood [Title/Abstract]) OR Juvenile[Title/Abstract]) OR newborn[Title/Abstract]) OR Offspring[Title/Abstract]) OR Suckling[Title/Abstract]) OR Toddlers[Title/Abstract]) OR Toddler[Title/Abstract]) OR Schoolchildren[Title/Abstract]) OR Pediatric[Title/Abstract]) OR Paediatrics[Title/Abstract]) OR Paediatric[Title/Abstract]) OR Paediatrics[Title/Abstract]) OR Girl[Title/Abstract]) OR Girls[Title/Abstract]) OR Boy[Title/Abstract]) OR Boys[Title/Abstract]) OR Youth [Title/Abstract]) OR Youths [Title/Abstract]) OR Preschool-child[Title/Abstract]) OR Minor[Title/Abstract]) OR infant[MeSH Terms]) OR child[MeSH Terms]) OR minors[MeSH Terms] |
| #3 | Search ((((((((((((((((Weaning[Title/Abstract]) OR Weaned[Title/Abstract]) OR Feeding[Title/Abstract]) OR Feed[Title/Abstract]) OR Family Foods[Title/Abstract]) OR Family Food[Title/Abstract]) OR Complementary feeding[Title/Abstract]) OR Complementary feedings[Title/Abstract]) OR Solid feeding[Title/Abstract]) OR Solid food[Title/Abstract]) OR Solid foods[Title/Abstract]) OR Food[Title/Abstract]) OR *Enteral food*[Title/Abstract]) OR Additional food[Title/Abstract]) OR Additional foods[Title/Abstract]) OR Additional feeding[Title/Abstract]) OR weaning[MeSH Terms] |
| #4 | Search (((((((((((((((((((((((((((((((((((Overweight[Title/Abstract]) OR Obesity[Title/Abstract]) OR Obese[Title/Abstract]) OR Obesitas[Title/Abstract]) OR Heavy[Title/Abstract]) OR Growth[Title/Abstract]) OR Body mass index[Title/Abstract]) OR BMI[Title/Abstract]) OR Body composition[Title/Abstract]) OR Adiposity[Title/Abstract]) OR adipositas[Title/Abstract]) OR Childhood obesity[Title/Abstract]) OR Excessive weight[Title/Abstract]) OR weight gain[Title/Abstract]) OR Weight-gain[Title/Abstract]) OR Over weight[Title/Abstract]) OR Weight change[Title/Abstract]) OR Overeating[Title/Abstract]) OR Overnutrition[Title/Abstract]) OR Body-Weight[Title/Abstract]) OR Body weight changes[Title/Abstract]) OR BMI-SDS[Title/Abstract]) OR SDS-BMI[Title/Abstract]) OR Body Weight[Title/Abstract]) OR Weight[Title/Abstract]) OR Childhood obesity[Title/Abstract]) OR Paediatric overweight[Title/Abstract]) OR Pediatric overweight[Title/Abstract]) OR Childhood overweight[Title/Abstract]) OR Body Weights and Measures[MeSH Terms]) OR Body Mass Index[MeSH Terms]) OR Obesity[MeSH Terms]) OR Overweight[MeSH Terms]) OR Pediatric obesity[MeSH Terms]) OR Adiposity[MeSH Terms]) OR Body Composition[MeSH Terms] |
| **Total** | **#1 AND #2 AND #3 AND #4** |

COCHRANE LIBARY

| Search | Terms |
| --- | --- |
| #1 | MeSH descriptor: [Infant, Premature] explode all trees |
| #2 | MeSH descriptor: [Premature Birth] explode all trees |
| #3 | MeSH descriptor: [Infant] explode all trees |
| #4 | MeSH descriptor: [Child] explode all trees |
| #5 | MeSH descriptor: [Overweight] explode all trees |
| #6 | MeSH descriptor: [Obesity] This term only |
| #7 | MeSH descriptor: [Adiposity] explode all trees |
| #8 | MeSH descriptor: [Pediatric Obesity] explode all trees |
| #9 | MeSH descriptor: [Overnutrition] explode all trees |
| #10 | MeSH descriptor: [Body Weights and Measures] explode all trees |
| #11 | MeSH descriptor: [Weaning] explode all trees |
| #12 | MeSH descriptor: [Child Nutritional Physiological Phenomena] explode all trees |
| #13 | ‘preterm’:ti,ab,kw or ‘premature’:ti,ab,kw or 'Prematurely':ti,ab,kw or ‘Prematurity’:ti,ab,kw |
| #14 | ‘Baby’:ti,ab,kw OR ‘Babies’:ti,ab,kw OR ‘Neonate’:ti,ab,kw OR ‘Neonates’:ti,ab,kw OR ‘Infant’:ti,ab,kw OR ‘Infants’:ti,ab,kw OR ‘Infancy’:ti,ab,kw OR ‘Child’:ti,ab,kw OR ‘Children’:ti,ab,kw OR ‘Childhood’:ti,ab,kw OR ‘Juvenile’:ti,ab,kw OR ‘Newborn’:ti,ab,kw OR ‘Offspring’:ti,ab,kw OR ‘Suckling’:ti,ab,kw OR ‘Toddler’:ti,ab,kw OR ‘Toddlers’:ti,ab,kw OR ‘Schoolchildren’:ti,ab,kw OR ‘Pediatric’:ti,ab,kw OR ‘Paediatrics’:ti,ab,kw OR ‘Paediatric’:ti,ab,kw OR ‘Paediatrics’:ti,ab,kw OR ‘Girl’:ti,ab,kw OR ‘Girls’:ti,ab,kw OR ‘Boy’:ti,ab,kw OR ‘Boys’:ti,ab,kw OR ‘Youth’:ti,ab,kw OR ‘Youths’:ti,ab,kw OR ‘Preschool-child’:ti,ab,kw OR ‘Minor’:ti,ab,kw |
| #15 | ‘Weaning’:ti,ab,kw OR ‘Weaned’:ti,ab,kw OR ‘Feeding’:ti,ab,kw OR ‘Feed’:ti,ab,kw OR ‘Complementary feeding’:ti,ab,kw OR ‘Family Foods’:ti,ab,kw OR ‘Family Food’:ti,ab,kw OR ‘Complementary feedings’:ti,ab,kw OR ‘Solid feeding’:ti,ab,kw OR ‘Solid food’:ti,ab,kw OR ‘Solid foods’:ti,ab,kw OR ‘Food’:ti,ab,kw OR ‘Foods’:ti,ab,kw OR ‘Diet’:ti,ab,kw OR ‘Intake’:ti,ab,kw OR ‘Infant feeding’:ti,ab,kw OR ‘Enteral foods’:ti,ab,kw OR ‘Additional food’:ti,ab,kw OR ‘Additional foods’:ti,ab,kw OR ‘Additional feeding’:ti,ab,kw OR ‘Nutrition’:ti,ab,kw |
| #16 | ‘Overweight’:ti,ab,kw OR ‘Obesity’:ti,ab,kw OR ‘Obese’:ti,ab,kw OR ‘Obesitas’:ti,ab,kw OR ‘Heavy’:ti,ab,kw OR ‘Growth’:ti,ab,kw OR ‘Body mass index’:ti,ab,kw OR ‘BMI’:ti,ab,kw OR ‘Queteletindex’:ti,ab,kw OR ‘Quetelet index’:ti,ab,kw OR ‘QI’:ti,ab,kw OR ‘Body composition’:ti,ab,kw OR ‘Adiposity’:ti,ab,kw OR ‘Adipositas’:ti,ab,kw OR ‘Childhood obesity’:ti,ab,kw OR ‘Excessive weight’:ti,ab,kw OR ‘weight gain’:ti,ab,kw OR ‘Weight-gain’:ti,ab,kw OR ‘Over weight’:ti,ab,kw OR ‘Weight change’:ti,ab,kw OR ‘Overeating’:ti,ab,kw OR ‘Overnutrition’:ti,ab,kw OR ‘Body-Weight’:ti,ab,kw OR ‘Body weight changes’:ti,ab,kw OR ‘BMI-SDS’:ti,ab,kw OR ‘SDS-BMI’:ti,ab,kw OR ‘Body Weight’:ti,ab,kw OR ‘Weight’:ti,ab,kw OR ‘Childhood obesity’:ti,ab,kw OR ‘Paediatric overweight’:ti,ab,kw OR ‘Pediatric overweight’:ti,ab,kw OR ‘Childhood overweight’:ti,ab,kw |
| #17 | #1 or #3 or #4 or #14 |
| #18 | #2 or #13 |
| #19 | #5 or #6 or #7 or #8 or #9 or #10 or #11 or #12 or #16 |
| **Total** | **#17 AND #18 AND #19 AND #15** |

EMBASE

| Search | Terms |
| --- | --- |
| #1 | ‘preterm’:ab,ti OR ‘premature’:ab,ti OR 'Prematurely':ab,ti OR ‘Prematurity’:ab,ti |
| #2 | ‘Baby’:ab,ti OR ‘Babies’:ab,ti OR ‘Neonate’:ab,ti OR ‘Neonates’:ab,ti OR ‘Infant’:ab,ti OR ‘Infants’:ab,ti OR ‘Infancy’:ab,ti OR ‘Child’:ab,ti OR ‘Children’:ab,ti OR ‘Childhood’:ab,ti OR ‘Juvenile’:ab,ti OR ‘Newborn’:ab,ti OR ‘Offspring’:ab,ti OR ‘Suckling’:ab,ti OR ‘Toddler’:ab,ti OR ‘Toddlers’:ab,ti OR ‘Schoolchildren’:ab,ti OR ‘Pediatric’:ab,ti OR ‘Paediatrics’:ab,ti OR ‘Paediatric’:ab,ti OR ‘Paediatrics’:ab,ti OR ‘Girl’:ab,ti OR ‘Girls’:ab,ti OR ‘Boy’:ab,ti OR ‘Boys’:ab,ti OR ‘Youth’:ab,ti OR ‘Youths’:ab,ti OR ‘Preschool-child’:ab,ti OR ‘Minor’:ab,ti OR 'child'/exp OR 'minor (person)'/exp |
| #4 | ‘Weaning’:ab,ti OR ‘Weaned’:ab,ti OR ‘Feeding’:ab,ti OR ‘Feed’:ab,ti OR ‘Family Foods’:ab,ti OR ‘Family Food’:ab,ti OR ‘Complementary feeding’:ab,ti OR ‘Complementary feedings’:ab,ti OR ‘Solid feeding’:ab,ti OR ‘Solid food’:ab,ti OR ‘Solid foods’:ab,ti OR ‘Food’:ab,ti OR ‘Foods’:ab,ti OR ‘Diet’:ab,ti OR ‘Intake’:ab,ti OR ‘Infant feeding’:ab,ti OR ‘Enteral foods’:ab,ti OR ‘Additional food’:ab,ti OR ‘Additional foods’:ab,ti OR ‘Additional feeding’:ab,ti OR ‘Nutrition’:ab,ti OR 'infant nutrition'/exp |
| #5 | ‘Overweight’:ab,ti OR ‘Obesity’:ab,ti OR ‘Obese’:ab,ti OR ‘Obesitas’:ab,ti OR ‘Heavy’:ab,ti OR ‘Growth’:ab,ti OR ‘Body mass index’:ab,ti OR ‘BMI’:ab,ti OR ‘Queteletindex’:ab,ti OR ‘Quetelet index’:ab,ti OR ‘QI’:ab,ti OR ‘Body composition’:ab,ti OR ‘Adiposity’:ab,ti OR ‘Adipositas’:ab,ti OR ‘Childhood obesity’:ab,ti OR ‘Excessive weight’:ab,ti OR ‘weight gain’:ab,ti OR ‘Weight-gain’:ab,ti OR ‘Over weight’:ab,ti OR ‘Weight change’:ab,ti OR ‘Overeating’:ab,ti OR ‘Overnutrition’:ab,ti OR ‘Body-Weight’:ab,ti OR ‘Body weight changes’:ab,ti OR ‘BMI-SDS’:ab,ti OR ‘SDS-BMI’:ab,ti OR ‘Body Weight’:ab,ti OR ‘Weight’:ab,ti OR ‘Childhood obesity’:ab,ti OR ‘Paediatric overweight’:ab,ti OR ‘Pediatric overweight’:ab,ti OR ‘Childhood overweight’:ab,ti or ‘childhood obesity’/exp OR 'obesity'/exp/mj |
| **Total** | **#1 AND #2 AND #3 AND #4 AND #5** |

Web of Science

| **Search** | **Terms** |
| --- | --- |
| #1 | TS=((Preterm) OR (Prematurely) OR (Premature) OR (Prematurity)) |
| #2 | TS=((Baby) OR (Babies) OR (Neonate) OR (Neonates) OR (Infant) OR (Infants) OR (Infancy) OR (Child) OR (Children) OR (Childhood ) OR (Juvenile) OR (newborn) OR (Offspring) OR (Suckling) OR (Toddlers) OR (Toddler) OR (Schoolchildren) OR (Pediatric) OR (Paediatrics) OR (Paediatric) OR (Paediatrics) OR (Girl) OR (Girls) OR (Boy) OR (Boys) OR (Youth ) OR (Youths) OR (Preschool-child) OR (Minor)) |
| #3 | TS=((Weaning) OR (Weaned) OR (Feeding) OR (Feed) OR (‘Complementary feeding’) OR (‘Family Foods’) OR (‘Family Food’) OR (‘Complementary feedings’) OR (‘Solid feeding’) OR (‘Solid food’) OR (‘Solid foods’) OR (Food) OR (Foods) OR (Diet) OR (Intake) OR (‘Infant feeding’) OR (‘Enteral food’) OR (‘Additional food’) OR (‘Additional foods’) OR (‘Additional feeding’) OR (Nutrition) OR (‘Supplementary Feeding’)) |
| #4 | TS=((Overweight) OR (Obesity) OR (Obese) OR (Obesitas) OR (Heavy) OR (Growth) OR (‘Body mass index’) OR (BMI) OR (Queteletindex) OR (Quetelet index) OR (QI) OR (‘Body composition’) OR (Adiposity) OR (adipositas) OR (‘Childhood obesity’) OR (‘Excessive weight’) OR (‘weight gain’) OR (‘Weight-gain’) OR (‘Over weight’) OR (‘Weight change’) OR (Overeating) OR (Overnutrition) OR (Body-Weight) OR (‘Body weight changes’) OR (BMI-SDS) OR (SDS-BMI) OR (‘Body Weight’) OR (Weight) OR (‘Childhood obesity’) OR (‘Paediatric overweight’) OR (‘Pediatric overweight’) OR (‘Childhood overweight’)) |
| **Total** | **#1 AND #2 AND #3 AND #4** |

Scopus

| **Search** | **Terms** |
| --- | --- |
| #1 | TITLE-ABS-KEY (Preterm) OR TITLE-ABS-KEY (Prematurely) OR TITLE-ABS-KEY (Premature) OR TITLE-ABS-KEY (Prematurity) |
| #2 | TITLE-ABS-KEY (Baby) OR TITLE-ABS-KEY (Babies) OR TITLE-ABS-KEY (Neonate) OR TITLE-ABS-KEY (Neonates) OR TITLE-ABS-KEY (Infant) OR TITLE-ABS-KEY (Infants) OR TITLE-ABS-KEY (Infancy) OR TITLE-ABS-KEY (Child) OR TITLE-ABS-KEY (Children) OR TITLE-ABS-KEY (Childhood ) OR TITLE-ABS-KEY (Juvenile) OR TITLE-ABS-KEY (newborn) OR TITLE-ABS-KEY (Offspring) OR TITLE-ABS-KEY (Suckling) OR TITLE-ABS-KEY (Toddlers) OR TITLE-ABS-KEY (Toddler) OR TITLE-ABS-KEY (Schoolchildren) OR TITLE-ABS-KEY (Pediatric) OR TITLE-ABS-KEY (Paediatrics) OR TITLE-ABS-KEY (Paediatric) OR TITLE-ABS-KEY (Paediatrics) OR TITLE-ABS-KEY (Girl) OR TITLE-ABS-KEY (Girls) OR TITLE-ABS-KEY (Boy) OR TITLE-ABS-KEY (Boys) OR TITLE-ABS-KEY (Youth ) OR TITLE-ABS-KEY (Youths) OR TITLE-ABS-KEY (Preschool-child) OR TITLE-ABS-KEY (Minor) |
| #3 | TITLE-ABS-KEY (Weaning) OR TITLE-ABS-KEY (Weaned) OR TITLE-ABS-KEY (Feeding) OR TITLE-ABS-KEY (Feed) OR TITLE-ABS-KEY (‘Family Foods’) OR TITLE-ABS-KEY (‘Family Food’) OR TITLE-ABS-KEY (‘Complementary feeding’) OR TITLE-ABS-KEY (‘Complementary feedings’) OR TITLE-ABS-KEY (‘Solid feeding’) OR TITLE-ABS-KEY (‘Solid food’) OR TITLE-ABS-KEY (‘Solid foods’) OR TITLE-ABS-KEY (Food) OR TITLE-ABS-KEY (Foods) OR TITLE-ABS-KEY (Diet) OR TITLE-ABS-KEY (Intake) OR TITLE-ABS-KEY (‘Infant feeding’) OR TITLE-ABS-KEY (‘Enteral food’) OR TITLE-ABS-KEY (‘Additional food’) OR TITLE-ABS-KEY (‘Additional foods’) OR TITLE-ABS-KEY (‘Additional feeding’) OR TITLE-ABS-KEY (Nutrition) OR TITLE-ABS-KEY (‘Supplementary Feeding’) |
| #4 | TITLE-ABS-KEY (Overweight) OR TITLE-ABS-KEY (Obesity) OR TITLE-ABS-KEY (Obese) OR TITLE-ABS-KEY (Obesitas) OR TITLE-ABS-KEY (Heavy) OR TITLE-ABS-KEY (Growth) OR TITLE-ABS-KEY (‘Body mass index’) OR TITLE-ABS-KEY (BMI) OR TITLE-ABS-KEY (Queteletindex) OR TITLE-ABS-KEY (‘Quetelet index’) OR TITLE-ABS-KEY (QI) OR TITLE-ABS-KEY (‘Body composition’) OR TITLE-ABS-KEY (Adiposity) OR TITLE-ABS-KEY (adipositas) OR TITLE-ABS-KEY (‘Childhood obesity’) OR TITLE-ABS-KEY (‘Excessive weight’) OR TITLE-ABS-KEY (‘weight gain’) OR TITLE-ABS-KEY (‘Weight-gain’) OR TITLE-ABS-KEY (‘Over weight’) OR TITLE-ABS-KEY (‘Weight change’) OR TITLE-ABS-KEY (Overeating) OR TITLE-ABS-KEY (Overnutrition) OR TITLE-ABS-KEY (Body-Weight) OR TITLE-ABS-KEY (‘Body weight changes’) OR TITLE-ABS-KEY (BMI-SDS) OR TITLE-ABS-KEY (SDS-BMI) OR TITLE-ABS-KEY (‘Body Weight’) OR TITLE-ABS-KEY (Weight) OR TITLE-ABS-KEY (‘Childhood obesity’) OR TITLE-ABS-KEY (‘Paediatric overweight’) OR TITLE-ABS-KEY (‘Pediatric overweight’) OR TITLE-ABS-KEY (‘Childhood overweight’) |
| **Total** | **#1 AND #2 AND #3 AND #4** |

CINAHL

| **Search** | **Terms** |
| --- | --- |
| #1 | TI Preterm OR TI Prematurely OR TI Premature OR TI Prematurity  AB Preterm OR AB Prematurely OR AB Premature OR AB Prematurity |
| #2 | TI Baby OR TI Babies OR TI Neonate OR TI Neonates OR TI Infant OR TI Infants OR TI Infancy OR TI Child OR TI Children OR TI Childhood OR TI Juvenile OR TI newborn OR TI Offspring OR TI Suckling OR TI Toddlers OR TI Toddler OR TI Schoolchildren OR TI Pediatric OR TI Paediatrics OR TI Paediatric OR TI Paediatrics OR TI Girl OR TI Girls OR TI Boy OR TI Boys OR TI Youth OR TI Youths OR TI Preschool-child OR TI Minor OR  AB Baby OR AB Babies OR AB Neonate OR AB Neonates OR AB Infant OR AB Infants OR AB Infancy OR AB Child OR AB Children OR AB Childhood OR AB Juvenile OR AB newborn OR AB Offspring OR AB Suckling OR AB Toddlers OR AB Toddler OR AB Schoolchildren OR AB Pediatric OR AB Paediatrics OR AB Paediatric OR AB Paediatrics OR AB Girl OR AB Girls OR AB Boy OR AB Boys OR AB Youth OR AB Youths OR AB Preschool-child OR AB Minor |
| #3 | TI Weaning OR TI Weaned OR TI Feeding OR TI Feed OR TI ‘Family Foods’ OR TI ‘Family Food’ OR TI ‘Complementary feeding’ OR TI ‘Complementary feedings’ OR TI ‘Solid feeding’ OR TI ‘Solid food’ OR TI ‘Solid foods’ OR TI Food OR TI Foods OR TI Diet OR TI Intake OR TI ‘Infant feeding’ OR TI ‘Enteral food’ OR TI ‘Additional food’ OR TI ‘Additional foods’ OR TI ‘Additional feeding’ OR TI Nutrition OR TI ‘Supplementary Feeding’  AB Weaning OR AB Weaned OR AB Feeding OR AB Feed OR AB ‘Family Foods’ OR AB ‘Family Food’ OR AB ‘Complementary feeding’ OR AB ‘Complementary feedings’ OR AB ‘Solid feeding’ OR AB ‘Solid food’ OR AB ‘Solid foods’ OR AB Food OR AB Foods OR AB Diet OR AB Intake OR AB ‘Infant feeding’ OR AB ‘Enteral food’ OR AB ‘Additional food’ OR AB ‘Additional foods’ OR AB ‘Additional feeding’ OR AB Nutrition OR AB ‘Supplementary Feeding’ |
| #4 | TI Overweight OR TI Obesity OR TI Obese OR TI Obesitas OR TI Heavy OR TI Growth OR TI ‘Body mass index’ OR TI BMI OR TI Queteletindex OR TI ‘Quetelet index’ OR TI QI OR TI ‘Body composition’ OR TI Adiposity OR TI adipositas OR TI ‘Childhood obesity’ OR TI ‘Excessive weight’ OR TI ‘weight gain’ OR TI ‘Weight-gain’ OR TI ‘Over weight’ OR TI ‘Weight change’ OR TI Overeating OR TI Overnutrition OR TI Body-Weight OR TI ‘Body weight changes’ OR TI BMI-SDS OR TI SDS-BMI OR TI ‘Body Weight’ OR TI Weight OR TI ‘Childhood obesity’ OR TI ‘Paediatric overweight’ OR TI ‘Pediatric overweight’ OR TI ‘Childhood overweight’  AB Overweight OR AB Obesity OR AB Obese OR AB Obesitas OR AB Heavy OR AB Growth OR AB ‘Body mass index’ OR AB BMI OR AB Queteletindex OR AB ‘Quetelet index’ OR AB QI OR AB ‘Body composition’ OR AB Adiposity OR AB adipositas OR AB ‘Childhood obesity’ OR AB ‘Excessive weight’ OR AB ‘weight gain’ OR AB ‘Weight-gain’ OR AB ‘Over weight’ OR AB ‘Weight change’ OR AB Overeating OR AB Overnutrition OR AB Body-Weight OR AB ‘Body weight changes’ OR AB BMI-SDS OR AB SDS-BMI OR AB ‘Body Weight’ OR AB Weight OR AB ‘Childhood obesity’ OR AB ‘Paediatric overweight’ OR AB ‘Pediatric overweight’ OR AB ‘Childhood overweight’ |
| **Total** | **#1 AND #2 AND #3 AND #4** |
